# Cell population-specific expression analysis of human cerebellum

**DOI:** 10.1186/1471-2164-13-610

**Published:** 2012-11-12

**Authors:** Alexandre Kuhn, Azad Kumar, Alexandra Beilina, Allissa Dillman, Mark R Cookson, Andrew B Singleton

**Affiliations:** 1 Laboratory of Neurogenetics, National Institute on Aging, National Institutes of Health, Bethesda, MD, USA; 2 Current address: Microfluidics Systems Biology, Institute for Materials Research and Engineering, A*STAR, 3 Research Link, Singapore, Singapore, 117602

**Keywords:** Genomics, Computational biology, Cerebellum, Gene expression, Aging, Astrocyte

## Abstract

**Background:**

Interpreting gene expression profiles obtained from heterogeneous samples can be difficult because bulk gene expression measures are not resolved to individual cell populations. We have recently devised Population-Specific Expression Analysis (PSEA), a statistical method that identifies individual cell types expressing genes of interest and achieves quantitative estimates of cell type-specific expression levels. This procedure makes use of marker gene expression and circumvents the need for additional experimental information like tissue composition.

**Results:**

To systematically assess the performance of statistical deconvolution, we applied PSEA to gene expression profiles from cerebellum tissue samples and compared with parallel, experimental separation methods. Owing to the particular histological organization of the cerebellum, we could obtain cellular expression data from in situ hybridization and laser-capture microdissection experiments and successfully validated computational predictions made with PSEA. Upon statistical deconvolution of whole tissue samples, we identified a set of transcripts showing age-related expression changes in the astrocyte population.

**Conclusions:**

PSEA can predict cell-type specific expression levels from tissues homogenates on a genome-wide scale. It thus represents a computational alternative to experimental separation methods and allowed us to identify age-related expression changes in the astrocytes of the cerebellum. These molecular changes might underlie important physiological modifications previously observed in the aging brain.

## Background

Over the last decade, genome-wide gene expression profiling has greatly improved upon the functional, molecular characterization of many tissues. For instance, our understanding of molecular changes occurring in particular human diseases has increased dramatically, in some cases leading to discovery of novel disease subtypes or more precise prognosis [1,2]. In practice, gene expression profiling studies are often performed on samples comprised of several distinct cell populations. In this case, expression levels of particular transcripts reflect their total abundance over all cells in the sample. Because gene expression measures in tissue homogenates are not resolved to individual cell populations, it can be difficult to reach conclusions regarding the cellular physiology of the individual cell populations. Moreover, heterogeneous samples often display variable composition. This can be the case for human tissue samples and typically results in additional variability of measured expression. For differential gene expression studies (e.g. disease versus control), this additional variability can decrease the probability of detecting expression changes and mask differences between sample groups. It can even lead to wrong estimates of expression changes in the case of systematic changes in tissue composition between test conditions.

Resolving gene expression to individual cell populations is of great relevance: It could allow the discovery of novel specific biomarkers or help identify small but important, cell type-specific expression differences (e.g. eQTLs) for instance. Experimental approaches to the problem of tissue heterogeneity include physically separating the cell population of interest from other cells in the sample, for instance using laser capture microdissection (LCM) or fluorescence-activated cell sorting. However, the applicability of such solutions greatly varies with the nature and availability of the tissue of interest: microdissecting individual cells spread throughout a tissue section can for instance be very time-consuming. Cell sorting, on the other hand, can be problematic with solid tissues. We have recently proposed Population-Specific Expression Analysis (PSEA), a computational method to deconvolute gene expression profiles measured in heterogeneous samples [3]. PSEA makes use of information on sample composition contained in the expression of population-specific marker genes and does not rely on additional experimental information. It can correct biased estimates of expression changes in the case of systematic differences in tissue composition between sample groups. This is important for the correct identification of changes occurring in the context of chronic diseases accompanied with specific histological changes (see [3] for an example of this effect in the context of neurodegeneration). Even in the absence of a systematic difference in tissue composition, many biological samples including brain, blood [4] and tumor samples [5] can display great variability and PSEA can improve the detectability of changes in gene expression when expression is contributed by cell populations whose abundance vary across samples.

Here, we applied PSEA on expression profiles measured from human cerebellum samples and deconvoluted the total expression level of each transcript into the contributions of several major cell populations, namely granule cells, Purkinje cells, astrocytes and oligodendrocytes. The strict spatial organization of cell populations in the cerebellum allowed us to verify deconvoluted gene expression patterns using the Allen Brain Atlas [6], a genome-wide atlas of gene expression obtained by colorimetric in situ hybridization (ISH). We also obtained gene expression measurements of laser-capture microdissected Purkinje cells and confirmed population-specific expression signals obtained with PSEA. We then used PSEA to perform differential expression analysis in a cell type specific manner and identified a set of genes with robust, age-related changes in astrocytic expression. These expression changes may be molecular mediators of important physiological modifications previously observed in the aging brain.

## Results

### Gene expression deconvolution using cell population-specific expression signals

To deconvolute gene expression measured from samples composed of several cell types, PSEA relies on the detection of a correlation between the total expression level of a gene of interest and the expression levels of marker genes, i.e. genes expressed in a single cell type only. Briefly, when the relative abundance of a given cell type varies from sample to sample, measured expression levels of genes expressed in this cell type co-vary with the size of this cell population. In particular, a cell type-specific marker gene thus specifically tracks variations in the size of an individual cell population since its expression will co-vary with the size of the corresponding expressing population and this one only. It follows that an arbitrary gene expressed in a particular population will correlate with marker genes expressed in this population as well. In practice, we used several marker genes for each population and average them to create single population-specific reference signals. We then identified populations contributing to total expression by detecting (possibly multiple) correlation between gene expression and a set of population-specific reference signals. Hence, the deconvolution problem is framed as a multiple linear regression problem and can be addressed with usual statistical methodology. The coefficients of this particular regression problem approximate (relative) population-specific expression levels [3]. This has two important consequences: first, it allows for the quantitative comparison of expression levels for various genes in a particular cell population. For instance if two genes are expressed in a given population but one of them is expressed in a second population as well, we can effectively factor out the expression in the second population and compare expression levels in the common population specifically. Second, this method can be used to test if the expression of a given gene is changed across different conditions within a single population. Notably, such a cell population-specific differential expression analysis can show increased sensitivity since it accounts for variability in sample composition.

### Cell population-specific analysis of gene expression profiles from cerebellum samples

**Figure 1:**
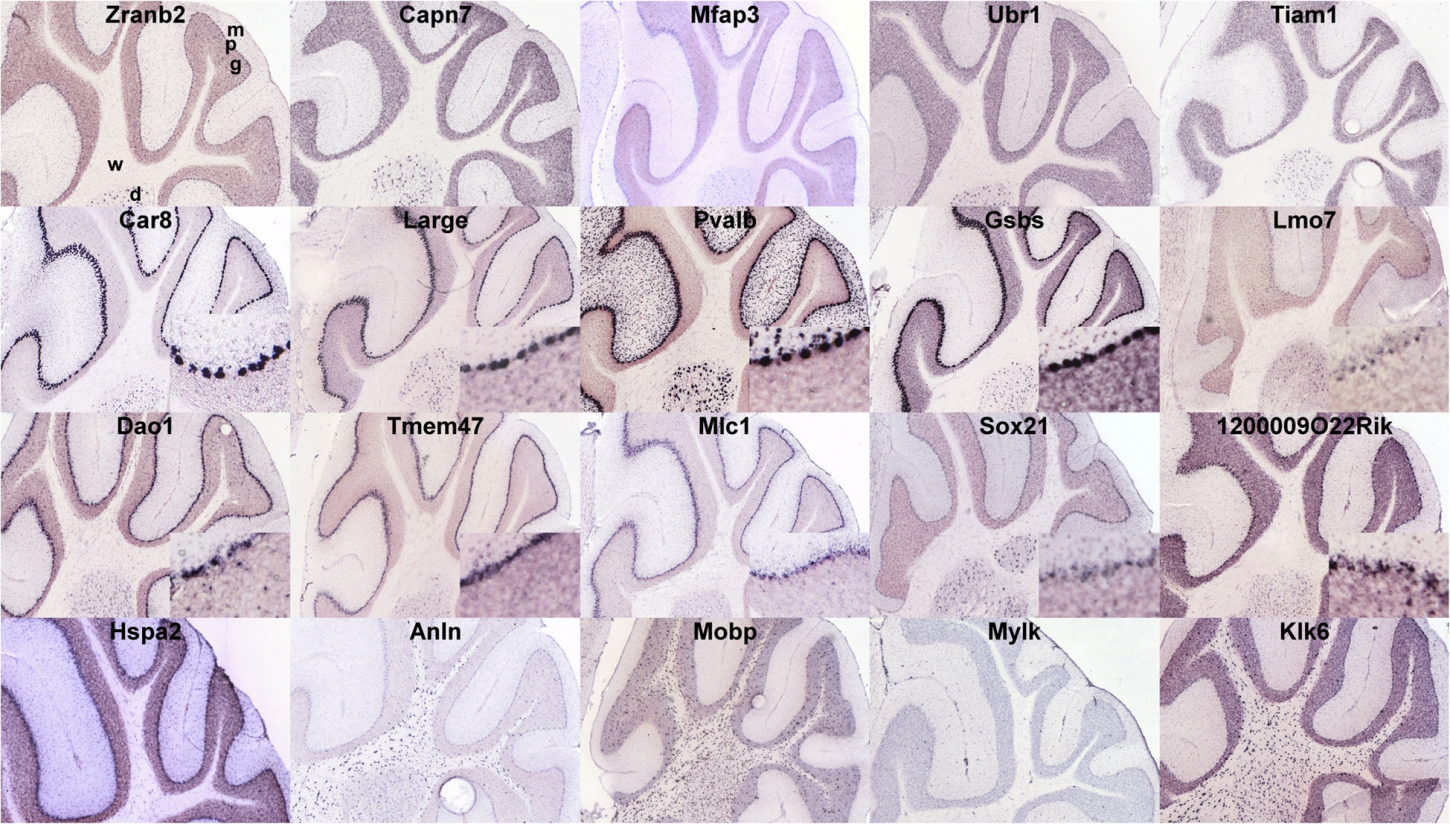
**Micrographs of colorimetric in situ hybridization experiments from the Allen Brain Atlas. **We show 5 top genes with predicted specific expression in granule cells (first row), Purkinje cells (second row), astrocytes (third row) and oligodendrocytes (fourth row). Each micrograph shows part of the mouse cerebellum (simple, ansiform and paramedian lobules, from left to right) on a sagittal section around level 1600. Genes significantly associated with a reference signal in the human gene expression data but without mouse ortholog, not tested or not detected in ISH experiments were not considered. Histological annotation in shown for the top-left micrograph: m: molecular layer, p: Purkinje cell layer, g: granular layer, w: white matter, d: deep nuclei.

We used PSEA to deconvolute gene expression profiles obtained from tissue samples dissected from human cerebellum. The cerebellum is composed of a layer of neural tissue, the cerebellar cortex, sitting on top of white matter containing myelinated axonal processes. A set of nuclei called deep cerebellar nuclei is embedded in the white matter, away from the cerebellar surface (histological features of the cerebellum are annotated on the top-left micrograph in Figure 1). The cerebellar cortex is distinctly divided in three layers: the granule cell layer is the deepest layer and is comprised of tiny excitatory cells called granule cells and various much rarer inhibitory cell types (including Golgi cells). Granule cells are densely packed and actually are the most numerous neurons in the brain [7]. On top of the granular layer sits a thin layer of very large, neuronal cells with spherical bodies called Purkinje cells. Finally, the outer most layer is comprised of neuronal processes including the axons of granule cells (called parallel fibers) and the dendritic trees of Purkinje cells, as well as inhibitory interneurons (stellate and basket cells). Astrocytes are found in all layers of the cerebellar cortex and are also abundant in the white matter (fibrous astrocytes) [8]. The Purkinje cell layer contains the cell bodies of specialized radial astrocytic cells called Bergmann glia that extend their processes into the molecular layer. Oligodendrocytes are abundant in the white matter but are common in the granule layer and also present in the other cortical layers [8].

We obtained gene expression profiles from small tissue fragments split from the cortical surface of larger frozen cerebellum samples (Additional file 1: Table S1). The assayed tissue samples were thus comprised of cortex and white matter but not of deep nuclei. These samples had been collected in the course of a previous study and were thus not primarily intended to test statistical deconvolution. Therefore, they allowed us to test the applicability of PSEA to a standard gene expression dataset from a human tissue-based expression study. We aimed to deconvolute total expression measured in these tissue fragments into contributions from the major cell populations in the sample, i.e. granule cells, Purkinje cells, astrocytes and oligodendrocytes. For each cell population, we selected two or three genes previously known to be specifically expressed in this cell type and averaged them to obtain population-specific reference signal (see Methods and Additional file 1: Table S3). For a given population, individual marker genes strongly co-varied from sample to sample, suggesting that their expression level paralleled the fraction of cells from this population in each sample (Additional file 2: Figure S1). For each gene assayed on the array (except marker genes used to construct reference signals), we then asked if the sample-to-sample variation in expression could be satisfactorily explained by the variations in the four selected cell populations and performed multiple regression of expression on the four population-specific reference signals. Because of the small number of samples considered here and to avoid overfitting we used a standard model selection procedure to select population-specific reference signals to include in the fit of each gene. Finally, the resulting gene expression models were characterized and genes whose expression variability could not be convincingly explained by the 4 reference signals (or a subset thereof) were eliminated (see Methods).

**Figure 2:**
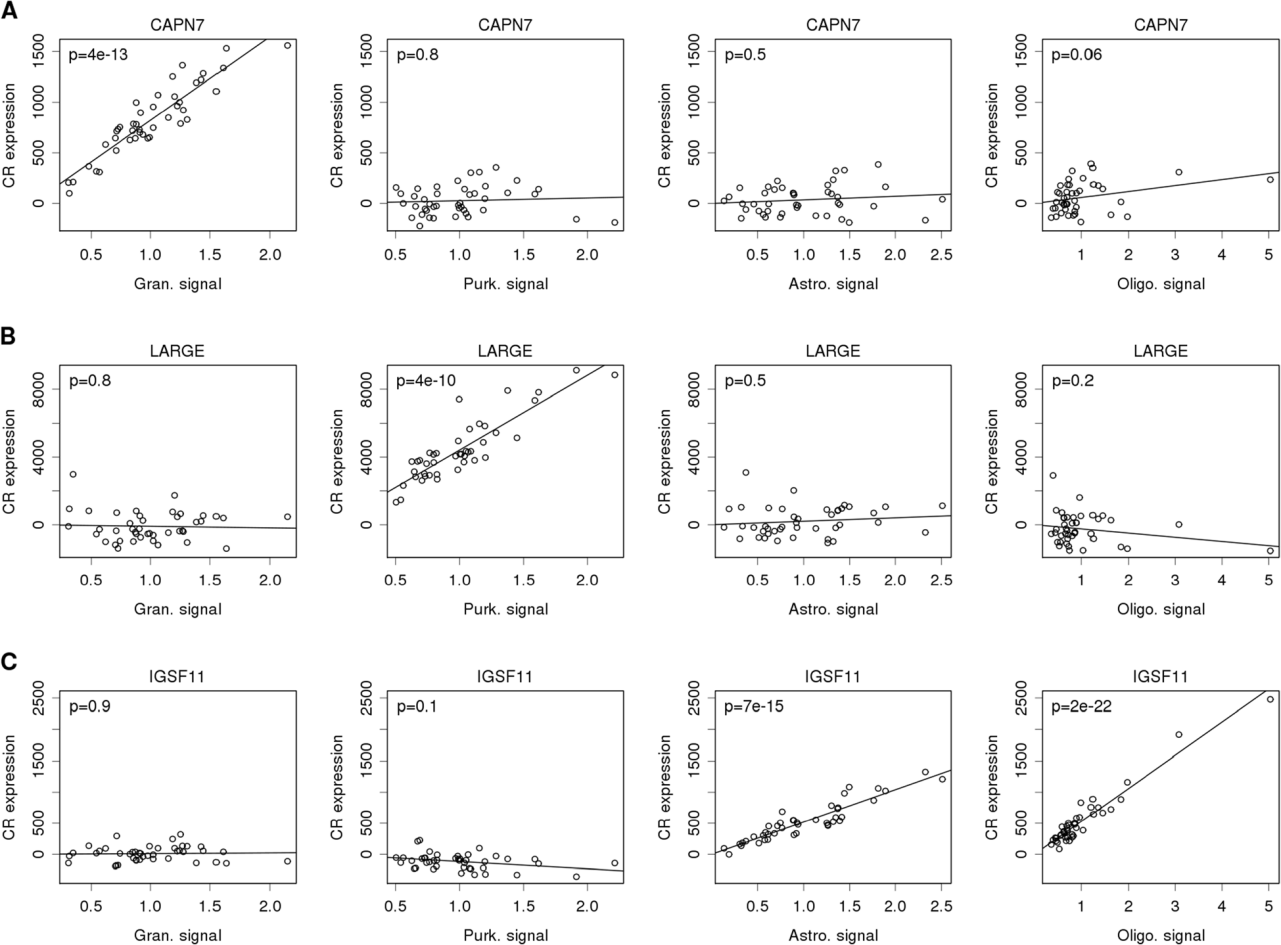
**Dependence of total gene expression on the population-specific reference signals.** For 3 selected genes, we show partial association with each of the 4 reference signals as a component-plus-residual plot. The slope of the partial regression is the relative population-specific expression level and the corresponding p-value is shown in the upper-left corner of each panel. **A**: Expression of CAPN7 is associated with the Purkinje reference signal; **B**: Expression of LARGE is associated with the Purkinje cell reference signal, **C**: Expression of IGSF11 is associated with both the astrocytic and the oligodendrocytic reference signals.

Figure 2 shows selected examples of genes and their association with population reference signals. The expression of CAPN7 (Figure 2A) was significantly associated with the granule cell reference signal, but not with any other reference signals. This suggested that CAPN7 was expressed in granule cells. Figure 2B shows that measured expression variations of LARGE was associated with variations in the Purkinje cell reference signal but not with the granular, astrocytic or oligodendrocytic signals. For most genes, total expression was associated with more than one reference signals. For example IGSF11 expression was found to be associated with both glial signals (Figure 2C).

Overall, we obtained statistical gene expression models for 5,952 probes (from a total of 22,184), with an average adjusted R^2^ of 0.69 (Additional file 3: Figure S2). The four reference signals were found to correlate differently to total expression: overall, the granule cell reference signal more largely accounted for total expression variability compared to the other 3 reference signals ($\text{average partial correlation coefficients}\;\bar{r_{G}}=0.65,\;\bar{r_{O}}=0.41,\;\bar{r_{A}}=0.4\;\text{and}\;\bar{r_{P}}=0.39$, for granule cell, oligodendrocyte, astrocyte and Purkinje cell reference signals, respectively; see also Additional file 4: Figure S3). The expression of most genes (5,645) was associated with the granule cell signal (Additional file 1: Table S4). Considering the histology of the cerebellum, we hypothesized that granule cells comprised the most abundant cell type in our samples, followed by astrocytes, oligodendrocytes and Purkinje cells. Although glial cells markedly outnumber Purkinje cells in the cerebellum [8], the latter have much larger cell bodies and it is difficult to estimate a priori the relative contribution of each cell type to total expression. We most often detected simultaneous contribution to total expression from the two neuronal cell populations and astrocytes (1,103 probes, Additional file 1: Table S4) and from the two neuronal cell populations alone (977 probes).

Notably, we detected a subset of genes with highly significant oligodendrocyte-specific expression (much larger than for any of the other 3 populations, see Additional file 4: Figure S3). Note the higher variability of the oligodendrocyte reference signal compared to the other 3 reference signals (see Additional file 2: Figure S1), which increased the detectability of oligodendrocyte expression (because in linear regression the sample variance of the coefficient is inversely proportional to the variance of the regressor). This underlines the beneficial role played by variability in sample composition for the statistical deconvolution scheme used here.

### In situ hybridization experiments support PSEA-predicted expression patterns

We used the Allen Brain Atlas (ABA) collection of in situ hybridization (ISH) experiments [6] to verify the cell type-specific gene expression predictions made with PSEA. The ABA project performed genome-wide ISH across the entire mouse brain, including the cerebellum. We hypothesized that gene expression patterns are largely conserved between human and mouse, paralleling the strong conservation of gene function across these two species [9]. Moreover, we reasoned that if human-based PSEA predictions showed systematic validation in mouse, we could rule out that the correspondence arose by chance. We identified genes predicted to have high specific expression in a single one of the four cell populations (see Additional file 5: Table S5, Additional file 6: Table S6, Additional file 7: Table S7, and Additional file 8: Table S8). We checked the expression of their murine orthologs (as defined by HomoloGene, NCBI) in the colorimetric mouse brain ISH experiments of the ABA project. Each row in Figure 1 shows micrographs of the cerebellum for ISH corresponding to the 5 first genes from each of the four lists of candidate genes with specific expression. Genes whose total expression was significantly associated with the granule cell signal are shown in the first row; as suggested by PSEA, they showed clear expression in the granule cell layer. The second row displays ISH for genes found to be significantly associated with the Purkinje cell signal. For all but one gene (Pvalb, see below), the staining was restricted to the thin layer of cells located between the granule cell and the molecular layers and corresponding to the Purkinje cell layer. At maximal resolution, typical bead-on-string patterns of individual Purkinje cells could be observed, as shown by the corresponding insets in the bottom right corner of each micrograph. Genes that showed strong association with the astrocyte signal (Figure 1, third row) often showed dense staining of the Purkinje cell layer as well. Closer inspection revealed microscopic staining patterns suggesting expression in Bergmann glia, as characterized by the staining of more numerous and smaller nuclei compared to Purkinje cells. For some genes, the staining included processes extending into the molecular layer (see e.g. Dao1) which is has been previously observed with gene expressed by Bergmann glia. Moreover, Dao1 and Mlc1 for instance have been shown previously to be expressed in astrocytes [10,11]. Small nuclei present in the granule layer were also stained by ISH, often but not always in conjunction with Bergmann glia staining, compatible with protoplasmic astrocytes. For instance Dao1 showed expression in both astrocytic subtypes (see Figure 1). Many genes with highly significant predicted expression in astrocytes, however, showed strong staining of Bergmann glia, suggesting that these cells accounted for a major proportion of the total astrocytic expression in cerebellum. Finally, the fourth row in Figure 1 shows genes predicted to be specifically expressed in oligodendrocytes: ISH probes for Anln, Mobp, and Klk6 specifically labeled numerous cells in the white matter, supporting the predicted specific oligodendrocyte expression. The majority of subsequent genes down the list of oligodendrocyte-specific expression also clearly revealed an oligodendrocyte staining pattern (not shown). Some genes, however, showed a very different staining pattern. Hspa2, for instance, showed faintly stained nuclei in the white matter but more prevalent staining in the Purkinje cell and molecular layers; Mylk on the other hand showed stained cells in every layer (Figure 1). We first checked that the sequences of the ISH probes (as well as the Illumina probes) were specific to the annotated gene transcripts. In line with the ABA experiment, Hspa2 has been previously reported to be expressed in mouse neurons and ependymal cells [12], whereas Mylk has been found to be expressed by smooth muscle cells [13]. This confirmed the expression patterns observed in the ABA atlas. We thus asked if these genes were expressed by cell types not taken into account in our deconvolution (possibly causing spurious association with the oliogdendrocyte reference signal) and tested for association between total expression and marker genes of additional minor cell types (see Methods). We did not find any significant association. Given that the transcriptional architecture of human MYLK appears to be more complex than its mouse ortholog (6 transcript variants for the human gene and 1 for mouse), we could not exclude that these genes might exhibit different cell specific regulation in the two species.

**Figure 3:**
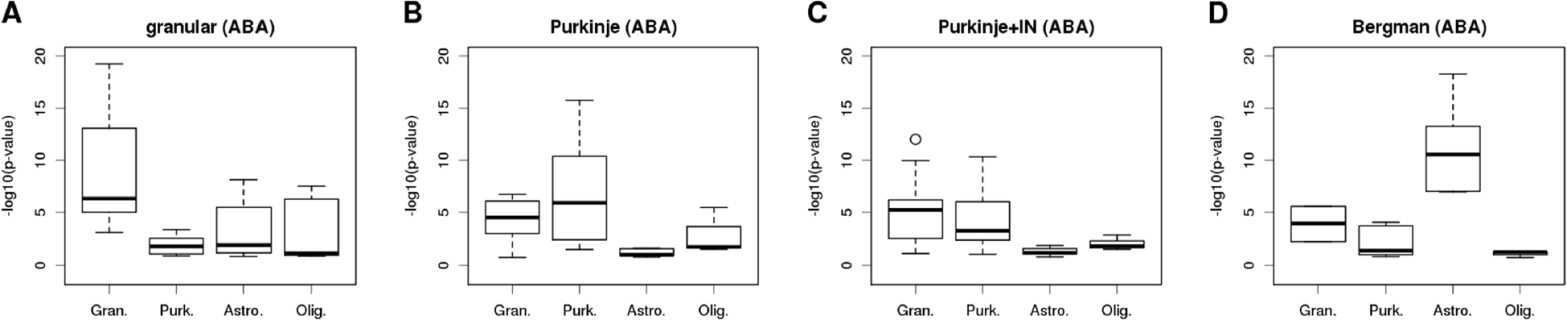
**Significance of PSEA-predicted, population-specific expression levels for 4 different groups of genes annotated by the ABA project. **Each panel corresponds to a group of genes found by the ABA project as specifically expressed in the granular layer (**A**), Purkinje cells (**B**), Purkinje cells and interneurons (IN) of the molecular layer (**C**), and Bergmann glia (**D**). Boxplots in each panel show the distributions of (−log_10_) p-values obtained with PSEA and associated with granular-specific, Purkinje-specific, astrocyte-specific or oligodendrocyte-specific expression levels. Boxplots in a single panel can represent a variable number of p-values since the number of reference signals accounted for in each gene expression model varies from gene to gene.

Based on their genome-wide ISH experiments, the ABA project annotated genes with specific expression in particular brain structures, including 50 genes specifically expressed in the granule cell layer, 15 genes specifically expressed in Purkinje cells, 30 genes expressed in Purkinje cells and in interneurons of the granular layer (Golgi cells), and 5 genes expressed by Bergmann glia. We asked if the corresponding expression models obtained with PSEA were in line with the ABA annotation (Figure 3). Genes annotated with granule cell-specific expression showed much smaller p-values associated with their expression in granule cells compared to the other three expression components (Figure 3A). Genes annotated with Purkinje cell expression were predicted by PSEA to have more significant expression in Purkinje cells (as well as some less significant expression in granule cells, Figure 3B). Interestingly, genes found by the ABA project to be expressed in Purkinje cells and interneurons of the granular layer showed a more significant predicted expression in Purkinje and granule cells compared to the other 2 cell populations (Figure 3C). It is reasonable to assume that the fraction of interneurons of the granular layer correlated very well with the general granule cell fraction. Therefore, gene expression from interneurons would be expected to be associated with the granule cell signal, which is what we observed here. Finally, the five genes annotated with Bergmann glia-specific expression showed much more significant predicted expression in astrocytes compared to the other 3 cell types (Figure 3D). In conclusion, these two-way comparisons with gene expression patterns inferred from ISH experiments showed that PSEA can identify cell type specific expression from bulk measurement of gene expression.

### Limitations to the detection of arbitrary co-expression patterns

The comparison of results from the statistical deconvolution and from ISH experiments also revealed particular circumstances in which gene expression was undetected by PSEA. Pvalb, for instance, was not only expressed in Purkinje cells as predicted in our analysis, but it was also expressed by cells of the molecular layer (Figure 1, second row). For the lack of appropriate markers, we could not account for cells of the molecular layer into in our deconvolution scheme (see Methods). Moreover, we suggest that the strong association of Pvalb expression with the Purkinje cell reference signal did not allow us to detect that another cell type was not accounted for in the analysis (see Methods). We also observed instances of failed detection in populations accounted for by corresponding reference signals. Zranb2, for instance, was predicted to be specifically expressed in granule cells but ISH revealed expression in Purkinje cells as well (Figure 1, first row). In general, we observed that the cell population whose expression was undetected corresponded to a population with lower average abundance (compared to the detected population, e.g. Purkinje cells versus granule cells for Zranb2). Together with the fact that overall PSEA more efficiently detected contribution from the more abundant population (i.e. granule cells, see above), we concluded that expression contribution by cell populations of lower abundance could effectively be masked by expression in a more abundant cell population. This masking effect prevented the systematic detection of arbitrary co-expression patterns. Possible reasons for this masking effect included the potential higher noise level present in reference signals of lower abundance populations, in line with the fact that hybridization-based array techniques have higher noise levels at low intensity signal. Thus the application of PSEA to gene expression profiles obtained with experimental methods that perform better at lower signal intensities (e.g. RNA-seq) might yield better detection of co-expression patterns.

### Comparison of statistical deconvolution and experimental microdissection

**Figure 4:**
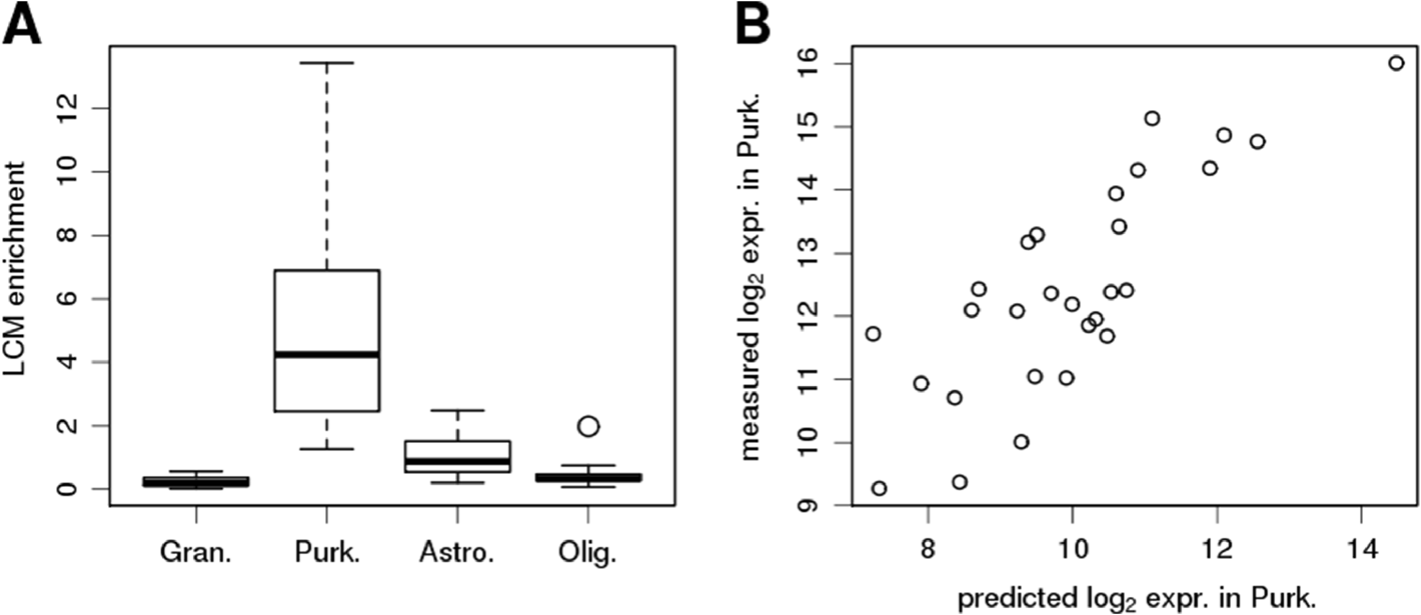
**Validation of Purkinje cell-specific expression detection with PSEA by comparison to gene expression measured in experimentally isolated Purkinje cells. A**: Ratio of gene expression levels measured in isolated Purkinje cells compared to whole tissue samples. Each boxplot corresponds to the top 30 genes predicted to be specifically expressed in one of the 4 cell populations. **B**: Predicted (log_2_) Purkinje cell-specific expression levels (x-axis) versus (log_2_) expression levels measured in experimentally isolated cells (y-axis).

To directly compare statistical deconvolution and experimental separation, we experimentally isolated a cell population from human cerebellum samples. We performed laser-capture microdissection (LCM) of Purkinje cells in a set of 100 samples and subjected them to gene expression profiling (see Methods). Given their large cell body size, Purkinje cells are relatively easy to separate from their surrounding tissue using LCM and thus allowed us to obtain a reasonably resolved expression profile. Moreover, they represent a minor cell population in our homogenate samples and thus provided an interesting test case of PSEA’s performance. We reasoned that genes correctly predicted to be significantly expressed in Purkinje cells by PSEA would show high expression in LCM samples, whereas genes predicted to be expressed in other cell population but not in Purkinje cells should show low expression in LCM samples. Figure 4A shows the expression enrichment obtained after LCM for four sets of top 30 genes predicted to be specifically expressed in one of the 4 cell populations (Additional file 5: Table S5, Additional file 6: Table S6, Additional file 7: Table S7, and Additional file 8: Table S8). Genes predicted to have highly specific expression in Purkinje cells showed high enrichment in LCM samples (mean enrichment 5.2) whereas genes with predicted expression in granule cells, astrocytes or oligodendrocytes (but not in Purkinje cells) showed low enrichment (mean enrichment 0.2, 1, and 0.4, respectively). The LCM enrichment measured for genes with predicted astrocytic expression was clearly higher than for genes with predicted expression in oligodendrocytes and granule cells. We suggest that this is the result of an experimental artefact and not a consequence of the deconvolution method: Purkinje cells and Bergman glia reside in close proximity, resulting in the potential contamination of LCM samples with astrocytes. This resulted in higher astrocyte enrichment compared to oligodendrocyte for instance since the latter reside in the white matter and are readily separated from Purkinje cells during the LCM procedure.

Furthermore, Purkinje cell-specific expression levels obtained with PSEA strongly correlated with the absolute expression measured in LCM samples (Figure 4B and Additional file 9: Figure S4). Thus, PSEA could successfully identify genes expressed in a minor cell population, as well as yield quantitative estimates of their specific expression levels.

### Detection of cell population-specific gene expression changes in cerebellum tissue samples

An important feature of PSEA is that it allows to test for differential expression within cell populations [3]. In contrast to a standard differential expression analysis performed on total expression levels, PSEA allows to resolve expression changes to particular cell populations. It can also yield more precise measures of expression change since it accounts for the variability (and potential systematic differences) in sample composition between experimental groups. We aimed to assess the effect of age on gene expression in the main cell types of the cerebellum (see Methods). Aging is accompanied with decreasing cognitive abilities and is the main risk factor for many neurodegenerative diseases. It is thus a major challenge in neuroscience to understand the cellular and molecular changes underlying normal, as well as pathological brain aging. We split our samples in two age groups (using median age 38 as threshold) and tested for differences in cell-type specific expression. We first focused on Purkinje cell expression because of the potential possibility to test candidate genes in our LCM dataset. We could not detect significant changes associated with this cell-type. However, we discovered a set of candidate genes associated with significant, specific expression changes in astrocytes (Table 1).

**Table 1:** Summary statistics for genes predicted to show an age-related, astrocyte-specific change in expression

**Probe ID**	**Expr (A)**	**p (A)**	**DE (dA)**	**p (dA)**	**ME**	**adj. R**^**2**^	**Gene symb.**	**Gene title, transcript variant**
ILMN_1653001	323	6.0 × 10^-9^	245	2.5 × 10^-6^	849	0.69	CABLES1	Cdk5 and Abl enzyme substrate 1
ILMN_1751904	802	6.1 × 10^-16^	−283	1.1 × 10^-5^	1009	0.86	EDNRB	endothelin receptor type B (EDNRB), transcript variant 2
ILMN_1813597	1792	7.6 × 10^-21^	−410	4.7 × 10^-5^	2333	0.93	C4orf18	chromosome 4 open reading frame 18 (C4orf18), transcript variant 1
ILMN_1796755	229	1.5 × 10^-9^	−119	7.4 × 10^-5^	604	0.76	ITGB5	integrin, beta 5 (ITGB5)
ILMN_1801377	863	6.2 × 10^-08^	−491	1.4 × 10^-4^	2077	0.80	SLC29A4	solute carrier family 29 (nucleoside transporters), member 4 (SLC29A4)
ILMN_1757180	67	5.8 × 10^-07^	41	2.1 × 10^-4^	303	0.76	WDR49	WD repeat domain 49 (WDR49)
ILMN_1732197	276	1.7 × 10^-12^	−98	2.3 × 10^-4^	586	0.86	MN1	meningioma (disrupted in balanced translocation) 1 (MN1)
ILMN_1685641	118	4.0 × 10^-6^	−75	5.4 × 10^-4^	438	0.79	BCHE	butyrylcholinesterase (BCHE)
ILMN_1754795	1459	4.5 × 10^-7^	−771	9.3 × 10^-4^	3588	0.76	FAT	FAT tumor suppressor homolog 1 (Drosophila) (FAT)
ILMN_1688868	1296	5.6 × 10^-16^	−314	1.1 × 10^-3^	1676	0.90	PPAP2B	phosphatidic acid phosphatase type 2B (PPAP2B), transcript variant 1
ILMN_1717636	1735	1.5 × 10^-15^	−492	1.1 × 10^-3^	2610	0.79	RGMA	RGM domain family, member A (RGMA)
ILMN_1800317	144	1.4 × 10^-11^	−48	1.3 × 10^-3^	364	0.84	WNT5A	wingless-type MMTV integration site family, member 5A (WNT5A)
ILMN_1738147	449	3.1 × 10^-6^	−260	1.5 × 10^-3^	996	0.56	NES	nestin (NES)
ILMN_1815102	1065	1.0 × 10^-9^	−416	1.5 × 10^-3^	1764	0.78	LCAT	lecithin-cholesterol acyltransferase (LCAT)
ILMN_1709486	1717	9.5 × 10^-19^	−321	2.3 × 10^-3^	2253	0.91	SRPX	sushi-repeat-containing protein, X-linked (SRPX)

The top 15 genes with the most significant expression changes are listed. Expr (A): astrocyte-specific expression level, p: p-value, DE (dA): astrocyte-specific differential expression, ME: mean expression, adj. R^2^: adjusted R^2^.

Many of the candidate genes with the most significant changes were found to play a role in the regulation of cell proliferation. EDNRB has been shown to have antiapoptotic effects in rat cerebellum [14] as well as to specifically mediate astrocyte proliferation [15]. The expression of FAT1, on the other hand, has been found to be reduced in astrocytic tumors [16]. Further genes whose function has been previously associated with the regulation of cell cycle progression included CABLES1 [17], ITGB5 [18], and MN1 [19]. These candidate changes might be particularly relevant in the context of previous observation showing increased relative number of astrocytes in the aging brain [20]. We also noted two genes encoding important metabolic enzymes and predicted to show age-related expression change. BCHE has been linked to the modulation of Alzheimer’s disease progression [21]. It encodes an enzyme with cholinesterase activity and was predicted here to be downregulated with age. In line with this prediction, Maetzler et al. have recently measured the corresponding enzymatic activity in serum and found that it decreased with age [22]. LCAT, on the other hand, encodes an enzyme with extracellular cholesterol esterifying activity. Cholesterol is a critical component of brain physiology and brain cholesterol level has been associated as a risk factor in Alzheimer’s disease [23]. Brain cholesterol level actually decreases with normal aging [24] and the hypothesized downregulation of LCAT expression predicted here might thus provide a new candidate mechanism underlying this metabolic change. In support of this hypothesis, LCAT has recently been shown to be expressed by astrocytes and to play a critical role in the maturation of brain high-density lipoproteins and cholesterol distribution [25].

**Figure 5:**
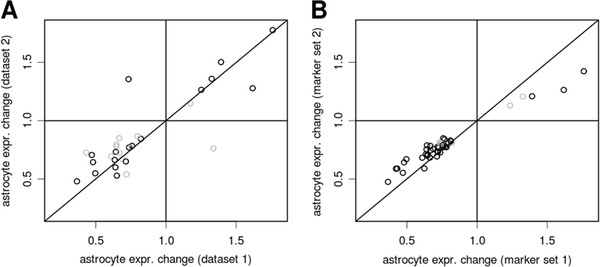
**Validation of age-related, astrocyte-specific expression changes in an independent dataset (A) and using an alternative astrocyte reference signal (B).A**: Out of 50 genes with astrocyte-specific expression change selected from our first dataset, 30 were found to show expression changes in a second, independent dataset. The plot shows astrocyte-specific fold change in expression detected in the first dataset (x-axis) and second dataset (y-axis). Black and gray open circles code for the significance of the p-values for the expression change detected in the second dataset (black: p<0.05, 18 genes; gray: p>0.05, 12 genes). Even though the corresponding p-values were not all nominally significant, all 30 genes supported gene expression changes upon statistical model selection. **B**: Out of 50 genes with astrocyte-specific expression change selected from our first analysis, 42 were found to show expression changes using an alternative, independent reference signal. Even though the corresponding p-values were not all nominally significant, all 42 genes supported gene expression changes upon statistical model selection.

We asked if these expression changes were robust and could be identified from another set of samples or using alternative marker genes for deconvolution. We applied PSEA on a set of gene expression profiles obtained from a separate collection of samples (n=57, median age=33, Additional file 1: Table S2). We used the same marker genes to construct population reference signals and applied PSEA on each candidate gene. Using the 50 genes with the most highly significant expression changes in the original dataset, we found that 30 showed evidence of astrocyte-specific, age-related expression changes in the second dataset (Figure 5A). The direction of change found in the two datasets was concordant for 28 genes and discordant for 2 genes, and the predicted fold change were clearly correlated across the 2 datasets. We then assessed the influence of the specific selection of marker genes on these results. We repeated the original analysis of the first sample set using an alternative astrocytic reference signal generated from 3 different, well-known astrocytic marker genes (Additional file 1: Table S3). Out of the top 50 genes originally showing age-related expression change in astrocytes, 42 supported an astrocyte-specific expression change based on the alternative reference signal. Furthermore, the predicted change estimated using either of the 2 alternative reference signals strongly correlated (Figure 5B).

Importantly, standard differential expression analysis did not highlight the particular gene set identified with PSEA. We tested the mean gene expression differences across sample groups and ordered all genes by decreasing evidence of differential expression. The top 50 genes predicted by PSEA to show astrocyte-specific changes had a median rank of 964.5 (mean rank: 4501) in the list obtained by standard analysis. The top 5 genes identified by PSEA showed a median rank of 573 (mean rank: 1134) in the list obtained by standard analysis. Thus, most genes identified here would have been missed using a standard differential analysis of bulk gene expression. In conclusion, statistical deconvolution identified a set of genes predicted to be expressed in astrocytes and showing robust, age-related changes in expression. These genes provide insights into potential molecular mechanisms underlying important physiological changes in the aging brain.

## Discussion

We applied a simple method to computationally deconvolute gene expression profiles from composite samples, based on the signal provided by marker genes. In contrast with previous methods, this analysis does not rely on additional external information like a separate assessment of sample composition [4,26-28] or the measure of gene expression profiles in isolated, homogeneous cell populations [29-33] (see also Venet et al. [34]). We performed deconvolution of gene expression measured from histologically complex, solid tissue samples and assessed the specificity of the predictions by comparing with in situ hybridization and laser-capture microdissection experiments. We showed that PSEA could correctly identify gene expression in specific cell types, including cell populations representing a minor fraction of the samples. Importantly, PSEA yields estimates of relative cell type-specific expression levels. It can be used to perform differential expression analysis and it is useful to assign gene expression changes to specific cell-types.

Several conditions are necessary for the application of PSEA. The method makes use of marker gene expression to build single population reference signals and track sample-to-sample variations in sample composition. To identify cell populations expressing a gene of interest, PSEA relies on the detection of statistical associations between variations in total gene expression and variations in population reference signals. As a first condition to the application of PSEA, we need to avail of marker genes for the cell types contributing to total expression. To date, marker genes have been identified for many cell types in numerous tissues (e.g. for the brain [35] and blood [36]). Indeed, they are invaluable research tools, allowing the identification of particular cells of interest via immunohistochemistry or used for driving cell-type specific transexpression for instance. Thus, the availability of marker genes should not be a limiting factor for the applicability of PSEA in general. If candidate markers are not available, their identification can be pursued experimentally, for instance by gene expression profiling of purified cell populations. Homogeneous cell populations can be isolated from tissues by histochemical stainings followed by microdissection or tissue dissociation and subsequent cell separation. Prior knowledge of cell surface markers or availability of a transgenic mouse line expressing a fluorescent reporter gene under the control of a cell-type specific promoter can facilitate the identification of further marker genes.

Candidate marker genes must be specific for a single cell type among all cells present in the samples. Expression in other cells absent from the samples of interest is of course allowable, extending the pool of genes that can be used as useful markers with PSEA. The suitability of candidate marker genes should be assessed thoroughly, including their specific expression in the cell population of interest and their expression range (see “Marker genes and population-specific reference signals” in Methods). Finally, prior knowledge of the histological composition of the tissue to be deconvoluted is important. Indeed, if the expression model obtained with PSEA does not account for a population that is actually contributing expression, estimates of population-specific expression levels for the populations that are accounted for in the model might be wrong (i.e. statistical “model misspecification”). Here we addressed this issue by taking into account all potential major contributors to gene expression in our samples. We also treated this problem a posteriori by filtering out genes whose statistical fit indicated that they might be expressed by another, unaccounted cell type (see Methods).

As a second general condition, heterogeneous samples should show variable composition. In fact, the more variable the abundance of a particular cell population, the larger the variability in the corresponding reference signal and the more precise the estimation of gene expression level. Here, we found that estimates of oligodendrocyte-specific expression levels had smaller standard error compared to other cell types. We suggest that this is due to larger variations in oligodendrocyte abundance, as reflected in the larger variability of the oligodendrocyte signal compared to other reference signals (Additional file 2: Figure S1). Gene expression profiles used for the present analysis were obtained following standard brain sample collection procedures. Our results indicate that, in this case, sample heterogeneity was sufficient to reliably estimate population-specific expression levels. However, the variations for several different cell populations should not show too much covariation. Correlation between independent regression variables results in the inflation of estimation errors and the impossibility to statistically separate contributions from individual populations (“collinearity”). In practice, this only occurs when regression variables show strong correlation (e.g. [37]) which is not what we observed between the four main neural populations in our cerebellum samples.

PSEA makes the important assumption that gene expression is proportional to the size of the corresponding population. This linear relationship between total expression and population size in collections of heterogeneous tissue samples has been verified in previous studies [29]. In the case of PSEA, it is particularly important that marker genes closely approximate this condition. In practice, we found that it was best met by selecting marker genes expressed in the medium range of the intensity scale. In principle, marker genes with high expression should yield better approximation of this assumption because their signal is relatively larger than the background of their expression measure. However, highly expressed genes could show saturated expression signals as measured by microarrays (e.g. GFAP, see Methods), which distorted the proportional relationship between expression and population abundance. Furthermore, the noise in marker gene expression measure decreased the sensitivity of PSEA (see Methods). Assuming that the noise was uncorrelated across different genes, we improved the quality of reference signals by averaging several marker genes. Nevertheless, we propose that the number of deconvoluted genes could be increased by using population reference signals with higher signal-to-noise ratios (see Methods). Although hybridization-based gene expression profiling has undergone spectacular improvements since its inception, it is not devoid of substantial measurement noise, particularly at low intensity signal. We hypothesize that the deconvolution of quantitatively more accurate expression profiles (such as those obtained from sequencing-based methods) might yield improved results.

## Conclusions

PSEA is widely applicable to studies dealing with heterogeneous samples, only relies on information contained in gene expression profiles and provides quantitative measures of cell population-specific expression levels. By direct comparison to in situ hybridization data and parallel laser-capture microdissection experiments, we have shown that PSEA can represent an efficient computational alternative to experimental separation methods. It has allowed us to identify expression changes in astrocytes that might underlie important physiological modifications previously observed in the aging brain.

## Methods

### Human cerebellum gene expression profiles and sample quality control

We have previously obtained gene expression profiles of human cerebellum samples from 150 individuals (NCBI GEO GSE15745) [38]. Gene expression profiling was performed in 3 batches. The first two batches were comprised of samples originating from a single collection site whereas the third batch was comprised of samples from several collection sites. We focused on the first two batches to ensure minimal technical variability between samples. We also thoroughfully assessed the quality of raw expression profiles and stringently filtered out samples deemed to be affected by technical variations in the two remaining batches: we first compared the values of each type of Illumina control probes across samples within each batch. One sample in the first batch had values for the standard Illumina “housekeeping” probes that were clearly different from the rest and it was eliminated. In the second batch, 4 samples were discarded because they had values for the standard Illumina “negative” and “labeling” control probes that differed from the rest, suggesting that a technical problem occurred during the preparation of labeled RNA for these samples. We then compared the raw distributions of gene expression values for each sample. In particular, we spotted outliers by comparing the sample median and standard deviation within each batch. Two samples of batch 2 were found to have outlying values for these two statistics and were eliminated. Finally, we looked at reference signals and spotted samples with outlying values for any of the reference signals. PSEA does not make any assumption on the distribution of population reference signals. In fact the larger the variability of the reference signal, the more precise the estimation of population-specific expression level. However, to avoid any single sample to have a large influence on the results of the regression, we eliminated samples with unusually high values for any of the reference signals. Two samples in batch 1 and 1 sample in batch 2 displayed large values for the oligodendrocyte reference signals and they were eliminated. A posteriori, we checked for the systematic influence of any single sample on the results of PSEA by inspecting the distributions of Cook’s distance [39] for all genes whose expression could be successfully deconvoluted (see below). In batch 2, 1 sample was found to have a median Cook’s distance that was clearly higher than all others. This suggested that this sample was influential for the fit of the majority of genes and it was discarded. In summary, we were left with 43 samples in batch 1 (Additional file 1: Table S1) and 57 in batch 2 (Additional file 1: Table S2). We applied PSEA in batch 1 and batch 2 separately and used batch 2 to assess the robustness our results obtained with batch 1.

To make full use of cell population variability and achieve better deconvolution, we performed population-specific expression (PSEA) within batches without prior normalization. This is not a prerequisite for the application of PSEA [3] but it improved the results obtained here because a single cell population (i.e. granule cells) was on average much more abundant that any other population. When this is the case (and this population contributes to the expression of a large number of genes), any normalization procedure will actually result in decreased gene expression variability (from this population). In other words, the variability in gene expression contributed by variations in the predominant population is confounded with sample to sample variation of technical origin and it is squelched by the normalization procedure since any normalization procedure is aimed at eliminating overall, sample to sample variability. However, with PSEA the larger the variability in the abundance of a particular population, the greater the sensitivity of specific expression detection in this population. Normalization will thus result in decreased sensitivity of gene expression detection for this population. In our cerebellum samples, most genes correlated with variations of the granule cell reference signal, suggesting that the expression variability of most genes was indeed dominated by variation in this population across samples. This variability was truly brought about by variability in sample composition and was greater than the variability of technical origin. This is demonstrated by the fact that reference signals for other cell populations did not co-vary with the granule cell signal (see Additional file 2: Figure S1), which would have been the case if expression variability was dominated by technical, array-to-array variability that would affect all genes similarly. Note finally that expression data should not be log transformed before PSEA (as opposed to a common practice in standard differential expression analysis) as the deconvolution method assumes a linear expression model where cell populations contributing expression add up, resulting in the “bulk” gene expression measured on the microarray [3].

### Marker genes and population-specific reference signals

We chose two to three well-known marker genes for each of the four cell populations that were expected to yield significant contributions to gene expression because of their abundance (granule cells, astrocytes and oligodendrocytes) or cellular size (Purkinje cells). We verified that the expression of marker genes from the same cell population were well correlated, indicating that their variability reflected the variable abundance of the corresponding cell type across samples. The marker genes and corresponding probes selected for our analysis are shown in Additional file 1: Table S3. Some well-known marker genes were dismissed because of their suspected saturated expression values (ZIC2 for granule cells, GFAP for astrocytes, FABP7 for Bergmann glia), which would violate the assumption of linearity between marker gene expression and population size. We also additionally checked the expression specificity of the selected marker genes in the Allen Brain Atlas and discarded GAD1 (Purkinje cell marker) because it showed strong expression in Golgi cells (in addition to Purkinje cells, see also Schilling et al. [40]). If the marker gene expression was measured by more than one probe on the array, we eliminated the probes whose signal was not clearly above background (e.g. AQP4).

Cell population reference signals were constructed as follows: First, each probe was given an equal weight by normalizing it to an average value of 1. When several probes reported expression of the same marker gene (i.e. for MBP) we averaged them to obtain a single marker gene expression measure. Finally, we averaged all marker gene expression measures within each cell population to obtain population-specific reference signals. We checked that the correlation between reference signals was moderate to avoid the problem of collinearity when performing regression. All pairwise correlations between the four reference signals were modest.

To test if we could improve deconvolution and separate known neuronal subpopulations further, we investigated the expression of genes recently found to be specifically expressed in interneurons of the molecular layer (ACCN1, GALNTL4, LYPD6, see Schilling et al. [40]). The resulting reference signal, however, highly correlated with the granule cell signal, preventing statistical separation of these two populations (because of collinearity). By inspection of ISH in the ABA, we noticed that GABRA6, a well-known granule cell markers might actually be weakly expressed in interneurons of the molecular layer as well. This suggested that the correlation of granule cell and interneuron reference signals was a consequence of the lack of gene markers with better specificity rather than co-abundance of the two cell populations across samples. Similarly, we also tried to separate radial glia (Bergmann glia) from other astrocytes (protoplasmic and fibrous). However, PPAP2B, a Bergmann glia marker, was highly correlated with AQP4 and GJA1 preventing us from statistically separating expression contributions from the different astrocyte subtypes. Since AQP4 and GJA1 are expressed by all astrocytes including Bergmann glia whereas PPAP2B is expressed specifically by Bergmann glia, this suggested that the astrocytic expression in our sample mostly originated from Bergmann glia.

### Statistical model building, fit characterization and implementation

Because of the limited number of samples in each batch and to avoid overfitting, we applied a variable selection method to find population reference signals that contributed to gene expression variability. To determine which reference signals to include in each gene expression model, we used a classical stepwise selection method based on Akaike’s AIC criterion [41]. We tested differential expression in specific cell populations by using the same variable selection strategy but allowing for an additional single interaction regressor (in addition to the 4 reference signals). Simultaneous detection of several population-specific changes was theoretically possible but we avoided it here because of the small sample size and high correlation between interaction regressors. PSEA was implemented with R [42]. We used the function step AIC (MASS package [43]) to perform statistical variable selection. The source code and data used for PSEA are provided as additional files (Additional file 10, Additional file 11, Additional file 12).

We characterized the statistical fits obtained by model building of each gene and models with a poor goodness-of-fit were discarded. We used the two following selection criteria: First, we discarded genes whose response variability was poorly explained the statistical model (adjusted R^2^ ≤ 0.5). Second, we interpreted large fitted intercepts as evidence of an expression source not represented by any of the four reference signals and we eliminated the corresponding genes from further consideration as well. The gene expression model used in PSEA implies a constant term (corresponding to the intercept in the regression) with an upper bound given by the background of expression measure (see Equation four in Kuhn et al. [3]). In practice, however, we observed that the fitted intercepts were moderately correlated with mean gene expression. This may be caused by noisy reference signals, which resulted in imperfect control of expression variability. Indeed, “error-in-variable” can lead to the intercept being biased toward the mean response (and thus overshooting the upper bound of the constant term in the expression model). Therefore and to avoid predominantly filtering out genes with large mean expression (which would result from the use of a fixed threshold on fitted intercepts), we used a relative criterion and eliminated genes with a ratio of fitted intercept over mean expression greater or equal to 0.5 (Additional file 3: Figure S2).

We also checked that assumptions underlying linear least-squares fitting were generally verified and we investigated error normality, error variance and linearity. Error distributions were deemed to be normal by comparing the sample distribution of studentized residuals with quantiles of the normal distribution (QQ plot), for a large number of fitted genes. Similarly, we examined studentized residuals versus fitted responses for a large number of gene expression models and concluded that most probe sets had constant error variance. A minority showed increasing error variance with increasing fitted expression values. This increase was modest and was deemed not to compromise ordinary least squares-based coefficient estimation. Finally, model linearity was checked by looking at partial residual plots. The majority of fitted genes did not show clear nonlinearity.

### Laser-capture microdissection and gene expression profiling of Purkinje cells

Frozen tissue samples of the cerebellum were obtained from 100 neurologically normal Caucasian subjects. Tissue was immersed in Shandon M-1 embedding matrix (Thermo Electron Corporation, Rockford, IL) and stored at −80°C until use. Cryostat sections (7–8 μm thick) were cut from frozen tissue samples using a Leica CM1900 cryostat (Leica, Houston, TX), and stored in pap jar (Evergreen, Los Angeles, CA) to avoid hydration. Before laser capture microdissection, brain sections were stained with Cresyl Violet (Ambion, Austin, TX) according to standard procedure. Given the notably unique morphology and position of Purkinje cells, this method of identification was sufficient to distinguish individual stained cells under the microscope. Laser-capture microdissection was performed with ArcturusXT microdissection system (Arcturus, Mountain View, CA). Purkinje cells were selected from the slide surface and captured on LCM Macro Caps. High-quality cellular RNA was recovered from the collected cells using PicoPureTM RNA isolation kit (Arcturus) and treated with RNase-free DNase (Qiagen, Valencia, CA). The quality of RNA was analyzed using an Agilent 2100 bioanalyzer (Agilent, Foster City, CA). Two rounds of amplification were carried out with the Ambion MessageAmp II aRNA kit.

Illumina human oligonucleotide arrays (HumanHT-12) were used according to the manufacturer's instructions, starting with 750 ng of amplified RNA for each sample. Array chips were scanned on an Illumina Bead array reader confocal scanner. The raw data can be obtained from NCBI GEO GSE37205. The Bioconductor package beadarray [44] was used to load raw Illumina gene expression data and to normalize them using robust multi-array average [45]. For comparison with gene expression measured in whole-tissue samples, we considered the set of probes that were present on the two array chips.

## Competing interests

The authors declare that they have no competing interests.

## Authors’ contributions

AKuhn conceived the study, performed the analyses and wrote the manuscript. AKumar and AB performed laser-capture microdissection and gene expression profiling experiments. AD helped with gene expression profiling experiments. ABS and MRC designed and directed the original gene expression profiling study and the laser-capture microdissection experiments. All authors read and approved the final manuscript.

## Supplementary Material

Additional file 1 **Table S1. **Sample characteristics for gene expression series 1. Series 1 was comprised of 43 samples. The minimal, 1st quartile, median, 3rd quartile and maximal age were 15, 20.5, 38, 48, and 72 years, respectively. Table S2: Sample characteristics for gene expression series 2. Series 2 was comprised of 57 samples. The minimal, 1st quartile, median, 3rd quartile and maximal age were 16, 24, 33, 45 and 58 years, respectively. Table S3: Marker genes used to construct reference signals for PSEA. Two different sets of astrocytic markers were used to generate 2 independent astrocytic reference signals. Set 2 was used to assess the robustness of astrocyte-specific changes detected with set 1. For some genes, we also used the following marker genes to test if expression could be detected in additional minor cell populations (see Results): DES (smooth muscle cells), CSPG4 (pericytes), P4HA1 (fibroblasts), PECAM1 (endothelial cells), CD37 (microglia) [35,46-55]. Table S4: Distribution of gene expression models obtained with PSEA upon statistical model building. G, P, A and O stand for the granular, Purkinje cell, astrocyte and oligodendrocyte reference signals, respectively. The third column indicates the average goodness-of-fit (as mean adjusted R^2^) for probes assigned a particular statistical model. Click here for file

Additional file 2 **Figure S1. **Expression levels of marker genes (left) and corresponding population-specific reference signals (right) for the granule (A), Purkinje (B), astrocytic (C) and oligodendrocytic (D) cell populations. For each row, the left panel shows the (log_2_) expression of marker genes across all samples. The right panel shows the reference signal obtained by averaging expression of the corresponding marker genes. The standard deviation of reference signals was 0.38 (granule cell), 0.36 (Purkinje cell), 0.55 (astrocyte), 0.8 (oligodendrocyte). Click here for file

Additional file 3 **Figure S2. **Characterization of gene expression models obtained upon statistical model building, for all genes on the microarray. Genes with better goodness-of-fit (higher adjusted R^2^) had smaller (relative) intercepts, in line with the hypothesized model for total expression (see Methods). Gray lines show the threshold criteria used for selecting expression models for further consideration (intercept/mean<0.5, adjusted R^2^>0.5). Click here for file

Additional file 4 **Figure S3. **Population-specific expression levels and associated p-values (for 5,952 genes passing fit quality criteria). Each volcano plot shows the (normalized) values for a particular model coefficient and corresponding (−log_10_) p-values (y-axis). Model coefficients were normalized by the average gene expression. A: intercept, B: granule cell-specific expression, C: Purkinje cell-specific expression, D: astrocyte-specific expression, E: oligodendrocyte-specific expression. Click here for file

Additional file 5 **Table S5. **Genes specifically associated with the granule cell reference signal. We selected genes with p-values for granule cell-specific expression at least 1000 times smaller than for any of the other 3 populations, and p-values for expression in these three populations greater than 0.001. Expr and p stand for coefficients (i.e. population-specific expression levels) and associated p-values, respectively. A, O, G and P indicate the corresponding reference signal: astrocyte, oligodendrocyte, granular and Purkinje, respectively. Click here for file

Additional file 6 **Table S6. **Genes specifically associated with the Purkinje cell reference signal. We selected genes with p-values for Purkinje cell-specific expression at least 1000 times smaller than for any of the other 3 populations, and p-values for expression in these three populations greater than 0.001. Expr and p stand for coefficients (i.e. population-specific expression levels) and associated p-values, respectively. A, O, G and P indicate the corresponding reference signal: astrocyte, oligodendrocyte, granular and Purkinje, respectively. Click here for file

Additional file 7 **Table S7. **Genes specifically associated with the astrocyte reference signal. We selected genes with p-values for astrocyte-specific expression at least 1000 times smaller than for any of the other 3 populations, and p-values for expression in these three populations greater than 0.001. Expr and p stand for coefficients (i.e. population-specific expression levels) and associated p-values, respectively. A, O, G and P indicate the corresponding reference signal: astrocyte, oligodendrocyte, granular and Purkinje, respectively. Click here for file

Additional file 8 **Table S8. **Genes specifically associated with the oligodendrocyte reference signal. We selected genes with p-values for oligodendrocyte-specific expression at least 1000 times smaller than for any of the other 3 populations, and p-values for expression in these three populations greater than 0.001. Expr and p stand for coefficients (i.e. population-specific expression levels) and associated p-values, respectively. A, O, G and P indicate the corresponding reference signal: astrocyte, oligodendrocyte, granular and Purkinje, respectively. Click here for file

Additional file 9 **Figure S4. **Predicted (log_2_) Purkinje cell-specific expression levels (x-axis) versus (log_2_) expression levels measured in experimentally isolated cells (y-axis) for all genes that obtained a non-negative, significant (p<0.05) Purkinje cell expression component by PSEA. Click here for file

Additional file 10 **Compressed (gzip) archive file (tar) containing computer code used for the PSEA analysis presented here. **The archive contains the main R code (psea.R) and associated functions (psea_f.R), sample information (param.txt), subject phenotypes (phenotypeInfo.txt) and microarray probe annotation file (GPL6104-20626.txt). Click here for file

Additional file 11 Compressed (zip) text file with the raw gene expression data used for the main analysis (i.e. subset of samples deposited in GEO GSE15745). Click here for file

Additional file 12 Compressed (zip) text file with the raw gene expression data used for validation (i.e. subset of samples deposited in GEO GSE15745). Click here for file
