# Supplementary material for: Cell population-specific expression analysis of human cerebellum
Source: BMC Genomics. 2012 Nov 12;13:610. doi: 10.1186/1471-2164-13-610 (PMC3561119; doi:10.1186/1471-2164-13-610)
Supplement: Additional file 1 — Table S1. Sample characteristics for gene expression series 1. Series 1 was comprised of 43 samples. The minimal, 1st quartile, median, 3rd quartile and maximal age were 15, 20.5, 38, 48, and 72 years, respectively. Table S2: Sample characteristics for gene expression series 2. Series 2 was comprised of 57 samples. The minimal, 1st quartile, median, 3rd quartile and maximal age were 16, 24, 33, 45 and 58 years, respectively. Table S3: Marker genes used to construct reference signals for PSEA. Two different sets of astrocytic markers were used to generate 2 independent astrocytic reference signals. Set 2 was used to assess the robustness of astrocyte-specific changes detected with set 1. For some genes, we also used the following marker genes to test if expression could be detected in additional minor cell populations (see Results): DES (smooth muscle cells), CSPG4 (pericytes), P4HA1 (fibroblasts), PECAM1 (endothelial cells), CD37 (microglia) [35,46-55]. Table S4: Distribution of gene expression models obtained with PSEA upon statistical model building. G, P, A and O stand for the granular, Purkinje cell, astrocyte and oligodendrocyte reference signals, respectively. The third column indicates the average goodness-of-fit (as mean adjusted R2) for probes assigned a particular statistical model. [file 1471-2164-13-610-S1.pdf]

Sample ID	Gender	Age	Cause of death	Post-mortem interval
1065	m	15	Multiple injuries	12
1076	m	17	accident, ruptured aneurysm	19
1078	f	17	Multiple injuries	12
1079	f	19	Toxic/metabolic (i.e. drug related)	16
1104	m	35	Multiple injuries	12
1114	m	31	Multiple injuries	15
1133	m	38	Cocaine intoxication	9
1158	m	16	cardiomegaly	15
1170	m	58	Cardiac arrythmia/endocarditis	24
1206	m	57	Multiple injuries	16
1226	m	23	drowning	21
1230	f	16	Multiple injuries	16
1496	f	53	cardiomyopathy	19
1498	m	48	Cardiovascular disease	20
1570	m	48	cardiovascular disease	14
1712	m	20	gunshot wound to the chest	8
1713	m	23	head and neck injuries	8
1715	f	33	asthma	17
1792	f	25	Multiple injuries	11
1795	f	49	Cardiovascular disease	23
1797	m	43	Multiple injuries	18
1825	m	48	cardiovascular disease	20
1028	m	39	compresional asphyxia and chest injuries	14
1037	m	19	Narcotic intoxication	11
1541	f	20	Head injuries	19
1827	f	39	Head injuries	9
1830	f	39	myocarditis	7
1847	m	57	Chest injuries complicating cardiovascular disease	16
1862	m	20	Multiple injuries	6
1865	m	55	cardiovascular disease	16
1866	m	32	Cardiovascular disease	19
1867	f	46	Multiple injuries	24
1902	m	43	Cardiac arrythmia	21
880	f	48	Cardiovascular disease	12
4263	m	61	Cardiac arrest	6
4789	f	72	Accident, exsanguination	19
5028	m	67	Multiple injuries	18
602	m	27	Accident, multiple injuries	15
604	m	43	Cardiovascular disease	15
813	f	30	Multiple injuries	14
819	m	18	chest injury	28
871	m	42	Toxic/metabolic (i.e. drug related)	19
879	m	21	Multiple injuries	13

Supplementary table 1

Sample ID	Gender	Age	Cause of death	Post-mortem interval
1105	m	16	Multiple injuries	17
1134	m	41	Cardiovascular disease	15
4549	m	58	Cardiovascular disease	17
1209	f	39	Chest and abdominal injuries	17
1260	m	42	Multiple injuries	8
1326	m	37	Cardiovascular disease	12
1465	m	17	Multiple injuries	4
1583	m	26	Multiple injuries	18
1846	f	20	Multiple injuries	9
1849	m	48	Head injuries complicating cardiovascular disease	21
1907	m	54	Multiple injuries	17
1940	m	57	Multiple injuries	20
4590	m	20	Dilated cardiomyopathy/morbid obesity	19
4540	m	25	Multiple injuries	23
4542	m	22	Multiple injuries	8
4543	m	28	Multiple injuries	13
4545	m	31	Cardiovascular disease	13
4593	m	33	Cardiac arrythmia	8
4598	m	45	Dilated cardiomyopathy	6
4636	f	43	Pulmonary embolism	19
4640	f	47	Pneumonia	5
4725	f	32	Hereditary Cardiovascular disease	17
4726	m	28	Multiple injuries	6
4729	m	24	Multiple injuries	10
4782	m	18	Head and chest injuries	17
4786	m	22	Multiple injuries	11
914	m	20	Accident, multiple injuries	18
927	m	17	Accident, multiple injuries	12
4841	f	42	Accident, multiple injuries	17
4842	m	47	Airway obstruction of food bolus	12
4903	f	31	Quetiapine/alcohol intoxication	5
4915	m	49	Cardiovascular disease	5
4916	m	19	Accident, drowning	5
4924	m	48	Cardiovascular disease	9
5024	m	56	Cardiovascular disease, DM	10
5078	m	48	Neck injuries/alcohol use	23
5079	m	33	Drowning complicated by alcohol intoxication	16
5087	m	44	Heroin intoxication/cocaine use	4
1454	f	47	combined drug intoxication	24
1502	m	29	Multiple injuries	19
1535	m	34	Abdominal injuries	16
1540	m	28	Multiple injuries	7
1544	m	32	Multiple injuries	12
1545	m	45	cardiomegaly with biventricular hypertrophy	20
1568	f	51	Pulmonary embolism	22
1571	f	18	Multiple injuries	8
1578	m	53	Cardiovascular disease	17
1584	f	18	Multiple injuries	15
1611	m	18	Hanging	11
1612	f	19	Multiple injuries	24
1613	f	41	Multiple drug intoxication	8
1614	f	27	Gunshot wound to the abdomen	18
1648	f	38	Mitral valve prolapse	23
1710	f	26	Cardiac tamponade	12
1909	m	40	Cardiovascular disease	20
1917	m	40	overdose / ?suicide	13
1936	m	46	Cardiovascular disease	13

Supplementary table 2

Cell type	Marker gene symbol	Illumina probe	Reference
Granule cell	GABRA6	ILMN_1798681	[46]
	NEUROD1	ILMN_1669425	[47]
Purkinje cell	PCP4	ILMN_1682326	[48]
	PCP2	ILMN_1720093	[49]
	CALB1	ILMN_1760199	[50]
Astrocyte (set 1)	APQ4	ILMN_1747683	[51]
	GJA1	ILMN_1727087	[52]
Astrocyte (set 2)	S100B	ILMN_1811590	[35]
	ALDH1L1	ILMN_1802167	[35]
	SLC1A3	ILMN_1738552	[35]
Oligodendrocyte	MBP	ILMN_1724599	[53]
		ILMN_1672660	
	MAG	ILMN_1803773	[54]
	MOG	ILMN_1737272	[55]

Supplementary table 3

Models	Counts	Mean adjusted R2
G+A+P	1103	0.68
G+P	977	0.64
G+A	772	0.65
G+P+O	735	0.74
P+A+G+O	701	0.76
G+O+A	641	0.73
O+G	466	0.72
G	250	0.62
P+O+A	79	0.73
A+P	69	0.66
O+A	52	0.81
O+P	34	0.76
O	28	0.8
P	27	0.61
A	18	0.75

Supplementary table 4
