# Supplementary material for: Cell population-specific expression analysis of human cerebellum
Source: BMC Genomics. 2012 Nov 12;13:610. doi: 10.1186/1471-2164-13-610 (PMC3561119; doi:10.1186/1471-2164-13-610)
Supplement: Additional file 2 — Figure S1. Expression levels of marker genes (left) and corresponding population-specific reference signals (right) for the granule (A), Purkinje (B), astrocytic (C) and oligodendrocytic (D) cell populations. For each row, the left panel shows the (log2) expression of marker genes across all samples. The right panel shows the reference signal obtained by averaging expression of the corresponding marker genes. The standard deviation of reference signals was 0.38 (granule cell), 0.36 (Purkinje cell), 0.55 (astrocyte), 0.8 (oligodendrocyte). [file 1471-2164-13-610-S2.doc]

Supplementary figure 1
