# Supplementary material for: Cell population-specific expression analysis of human cerebellum
Source: BMC Genomics. 2012 Nov 12;13:610. doi: 10.1186/1471-2164-13-610 (PMC3561119; doi:10.1186/1471-2164-13-610)
Supplement: Additional file 3 — Figure S2. Characterization of gene expression models obtained upon statistical model building, for all genes on the microarray. Genes with better goodness-of-fit (higher adjusted R2) had smaller (relative) intercepts, in line with the hypothesized model for total expression (see Methods). Gray lines show the threshold criteria used for selecting expression models for further consideration (intercept/mean<0.5, adjusted R2>0.5). [file 1471-2164-13-610-S3.doc]

Supplementary figure 2
