# Supplementary material for: Cell population-specific expression analysis of human cerebellum
Source: BMC Genomics. 2012 Nov 12;13:610. doi: 10.1186/1471-2164-13-610 (PMC3561119; doi:10.1186/1471-2164-13-610)
Supplement: Additional file 4 — Figure S3. Population-specific expression levels and associated p-values (for 5,952 genes passing fit quality criteria). Each volcano plot shows the (normalized) values for a particular model coefficient and corresponding (−log10) p-values (y-axis). Model coefficients were normalized by the average gene expression. A: intercept, B: granule cell-specific expression, C: Purkinje cell-specific expression, D: astrocyte-specific expression, E: oligodendrocyte-specific expression. [file 1471-2164-13-610-S4.doc]

Supplementary figure 3
