# Supplementary material for: Cell population-specific expression analysis of human cerebellum
Source: BMC Genomics. 2012 Nov 12;13:610. doi: 10.1186/1471-2164-13-610 (PMC3561119; doi:10.1186/1471-2164-13-610)
Supplement: Additional file 9 — Figure S4. Predicted (log2) Purkinje cell-specific expression levels (x-axis) versus (log2) expression levels measured in experimentally isolated cells (y-axis) for all genes that obtained a non-negative, significant (p<0.05) Purkinje cell expression component by PSEA. [file 1471-2164-13-610-S9.doc]

Supplementary figure 4
